# An audit of marker placement in stereotactic guided biopsy

**DOI:** 10.1186/bcr3788

**Published:** 2015-11-05

**Authors:** Jane Prady, Lucy Hill, Alison Gray, Alison Gilchrist

**Affiliations:** 1 South East of Scotland Breast Screening Service, Edinburgh, UK; 2 Queen Margaret University, Edinburgh, UK; 3 British Society of Breast Radiology, Edinburgh, UK

## Introduction

Anecdotal evidence suggests that there is a greater incidence of marker migration using large volume sampling techniques in stereotactic guided breast biopsies.

## Methods

Prospective study of 130 biopsies with markers done between June and December 2014. Markers more than 10 mm from the target lesion were considered migrated. The aim of the audit was to quantify the number of markers migrating, distance and direction of migration and conditions under which markers migrate.

## Results

A total of 12.3 % had migrated markers: 10.7 % from use of the Bard Encor system and 1.5 % from use of the Bard Vacora system. The greatest marker migration occurred using a latero-medial approach. The majority of migrated markers were deeper than the target lesion. Marker migration was significantly greater using the Encor system within lucent breast tissue. Firstly, further audit is required incorporating lesion size, routine vacuuming of the cavity before deployment of marker, specific sequencing of marker films, correlation of compressed breast thickness and target depth, clinical impact of marker migration and possible development of expanding marker. Secondly, the breast screening service should provide guidelines regarding distances, thresholds and targets for marker migration.

## Conclusion

This audit found that marker migration occurred predominantly within lucent breast tissue and using the latero-medial approach when using the Bard Encor system.

